# New records and ecological insights of *Spondylus limbatus* (Bivalvia, Spondylidae) from the Panamic Province

**DOI:** 10.3897/BDJ.14.e180522

**Published:** 2026-02-13

**Authors:** Julio A. Cotom-Nimatuj, Jesús Emilio Michel-Morfín, Giovanni Bassey-Fallas, Johanna Segovia, Ashley E. Sharpe, Osmar Sandino, Pedro Jiménez-Prado, Antonio Jaramillo-Arango, Valentín Mogollón-Ávila, Luis Gómez-Gastelum, Rodrigo Esparza-López, Richard Lunniss, Philippe Béarez

**Affiliations:** 1 BioArch (UMR 7209), Muséum national d'Histoire naturelle/CNRS, Paris, France BioArch (UMR 7209), Muséum national d'Histoire naturelle/CNRS Paris France; 2 Centro Universitario de Ciencias Biológicas y Agropecuarias, Universidad de Guadalajara, Guadalajara, Mexico Centro Universitario de Ciencias Biológicas y Agropecuarias, Universidad de Guadalajara Guadalajara Mexico; 3 Laboratorio de Biología, Universidad de Costa Rica, Sede de Guanacaste, Liberia, Costa Rica Laboratorio de Biología, Universidad de Costa Rica, Sede de Guanacaste Liberia Costa Rica; 4 Universidad de El Salvador, San Salvador, El Salvador Universidad de El Salvador San Salvador El Salvador; 5 Coiba Scientific Station (Coiba-AIP), Panama City, Panama Coiba Scientific Station (Coiba-AIP) Panama City Panama; 6 Center for Tropical Paleoecology and Archaeology, Smithsonian Tropical Research Institute, Balboa-Ancon, Panama City, Panama Center for Tropical Paleoecology and Archaeology, Smithsonian Tropical Research Institute, Balboa-Ancon Panama City Panama; 7 Fauna & Flora International, Managua, Nicaragua Fauna & Flora International Managua Nicaragua; 8 The Nature Conservancy, Quito, Ecuador The Nature Conservancy Quito Ecuador; 9 Instituto Colombiano de Antropología e Historia, Bogotá, Colombia Instituto Colombiano de Antropología e Historia Bogotá Colombia; 10 Universidad Nacional Federico Villarreal, Lima, Peru Universidad Nacional Federico Villarreal Lima Peru; 11 Universidad de Guadalajara, Guadalajara, Mexico Universidad de Guadalajara Guadalajara Mexico; 12 Centro de Estudios Arqueológicos, El Colegio de Michoacán, A. C., La Piedad de Cabadas, Mexico Centro de Estudios Arqueológicos, El Colegio de Michoacán, A. C. La Piedad de Cabadas Mexico; 13 Universidad Técnica de Manabí, Portoviejo, Ecuador Universidad Técnica de Manabí Portoviejo Ecuador

**Keywords:** Mollusca, Spondylidae, distribution records, reference collection, rocky reef habitats, thorny oyster, Tropical Eastern Pacific

## Abstract

**Background:**

Although *Spondylus limbatus* is a fishery resource in several Latin American countries and has been widely reported throughout the Panamic Province, gaps remain in the knowledge of its distribution and ecology. This bivalve has been exploited since pre-Hispanic times, although the specific harvesting grounds are still uncertain. To contribute to malacological issues of both archaeological and contemporary interest, we established a reference collection that will serve as a basis for future studies, particularly geochemical analyses. Sampling consisted of recovering approximately four individuals per locality, obtained mainly from local food consumption, while recording information on their provenance and collection environment, amongst other data. In total, 180 individuals were recovered from shallow waters at 72 localities distributed across eight countries of the region. Collection sites included open coasts, gulf and bays that may comprise islands and shoals.

**New information:**

This work documents new localities in areas where previous information was scarce or absent, such Jalisco in Mexico and El Salvador, Nicaragua and Panama, thereby significantly expanding current knowledge of the distribution of this species.

## Introduction

Four species of the genus *Spondylus* (Bivalvia, Spondylidae) are distributed throughout the Panamic Province, ranging from the Pacific coast of the Baja California Peninsula, Mexico, to northern Peru, including some oceanic islands, such as Isla del Coco (Skoglund and Mulliner 1996, Coan and Valentich-Scott 2012, Lodeiros et al. 2016). Amongst them, *Spondylus limbatus* is noteworthy because, after its larval stage, it attaches most of its right valve to hard substrates, such as rocks and shells, in rocky and coral reef habitats of warm waters, from the lower intertidal zone down to depths of 55 m (Lamprell 1987, Soria et al. 2012, Lodeiros et al. 2016). Genetic and spatial analyses suggest that its larvae may disperse, depending on factors such as tidal regime and the absence of natural barriers, over distances exceeding ~ 100 km from the fertilisation site (Soria et al. 2012). It is also the largest Panamic species, with shells reaching up to 25 cm in length, characterised by a wide internal margin with purple to orange hues, a large adductor muscle and short spines when present (Skoglund and Mulliner 1996, Coan and Valentich-Scott 2012, Lodeiros et al. 2016).

Although several studies have been conducted on this species, primarily in the Baja California Peninsula (Cudney-Bueno and Rowell 2008, Soria et al. 2012, Villalejo-Fuerte et al. 2023) and in Ecuador (Fabara 2008, Mackensen and Brey 2013, Loor et al. 2016), its distribution and ecology in other parts of the Province remain poorly understood. This is partly because the species is most often mentioned in general faunal lists rather than in targeted ecological studies. While multiple localities document its wide distribution, its pattern is not uniform (Carter 2022). Consequently, its presence may be underestimated, with additional localities not yet reported in scientific literature, but well known to fishers who collect the species both for subsistence and local trade, given its fishery value (Poutiers 1995, Barraza 2006, Villalejo-Fuerte et al. 2020, INCOPESCA 2023).

Beyond its current use, *Spondylus limbatus*, together with *Spondylus crassisquama*, was employed by various pre-Hispanic cultures as ritual offerings or for the manufacture of ornaments (Hocqhenghem 2010, Solís del Vecchio and Herrera Villalobos 2015, Jaramillo Arango 2017, Castillo Velasco and Velázquez Castro 2020, Eeckhout et al. 2023). This raises questions regarding the original collection sites, since much of the archaeological evidence comes from contexts located far from the species’ natural distribution (Murra 1982, Marcos 1986, Temple Sánchez-Garavito and Velázquez Castro 2003, Carter 2022). However, geochemical studies that could test these hypotheses have not yet been conducted for this species, unlike other taxa in different cultural regions (Mouchi et al. 2018, Mouchi et al. 2020).

In this context, the present article aims to report the results of *Spondylus limbatus* collections and the establishment of a dry reference collection that provides new data on the distribution and ecology of the species. This collection is also intended for future geochemical research. Ultimately, it has the potential to contribute to archaeomalacological studies and, in the long term, to serve as a historical record of the current environmental conditions of this fishery resource in the Tropical Eastern Pacific Ocean.

## Materials and methods

### Selection of sampling sites

Shells of *Spondylus limbatus* were collected from multiple localities, distributed along the Panamic Province between 2022 and 2023. The selection of these localities was based on three criteria: (1) sites reported in literature; (2) areas with evidence of pre-Hispanic exploitation (Temple Sánchez-Garavito and Velázquez Castro 2003); and (3) new sites suggested by local fishers, where no previous records of this species existed. A distance of 150–200 km was established between sampling localities, considering the potential geochemical and molecular differences in shells. This interval was proposed considering the potential dispersal distances of larvae and/or the presence of geomorphological features (e.g. open coasts, gulfs or bays) that may lead to environmental and ecosystem changes, potentially influencing the geochemical signatures to be recorded in the future within the exoskeleton of this mollusc. It should be noted that no collections were conducted within protected areas.

### Sampling strategy

To minimise impacts on *Spondylus* populations beyond local exploitation, the sampling strategy was limited to collecting only post-consumption discards, with an average of four specimens per locality. When possible, amongst the available shells, individuals showing variation in size, shape and colour were selected from the samples provided by fishers. Importantly, no direct extractions were performed for this study and no live animals were handled; therefore, no ethical approval was required. The collection was supplemented with specimens gathered by other researchers between 1994 and 2024, who donated them for this study. Only specimens with clear and precise information regarding collection locality, date, depth and other relevant data were included. When the collection site was not clearly known, the fisher was asked to indicate the approximate capture point on a map and those coordinates were assigned as the specimen’s locality of origin. Except for three localities, no beach-collected material was included. The fishers authorised the use of the information related to the sample collection of this research.

### Specimen database and species identification

The provenance information of all specimens was recorded in a database. Each individual was photographed, measured (umbo-to-ventral margin height, anteroposterior length and thickness), using an electronic digital caliper with a precision of ± 0.1 mm and weighed using a digital balance with a precision of ± 1 g. Each specimen was assigned a preliminary unique code, even in cases where two valves formed a pair. Once incorporated into the Malacology Collections of the Zooteca (Zoothèque) at the Muséum national d’Histoire naturelle (MNHN), each specimen will be assigned a definitive catalogue number. Species identification was carried out, based on morphological characteristics, compared with the descriptions available in Keen (1971), Skoglund and Mulliner (1996), Coan and Valentich-Scott (2012) and Lodeiros et al. (2016). This phase was conducted at the BioArchéologie, Interactions Sociétés Environnements laboratory (UMR7209 – BioArch) of the MNHN in Paris, France. In doubtful cases, specimens were compared with *Spondylus* reference material preserved in the malacology section of the MNHN.

## Data resources

At present, access to the MNHN databases remains temporarily unavailable due to a recent cyberattack affecting the institution's informatics infrastructure. Consequently, it has not yet been possible to deposit the dataset in public repositories such as GBIF. The data will be uploaded once access is restored.

The data presented in this study are available upon request from the corresponding author, as they form part of the first author’s ongoing doctoral dissertation.

## Taxon treatments

### Spondylus limbatus

G. B. Sowerby II, 1847

DB8150FF-5C1A-5389-875D-26C16345A99C

https://www.molluscabase.org/aphia.php?p=taxdetails&id=207886

#### Description

According to Keen (1971), Poutiers (1995), Coan and Valentich-Scott (2012) and Lodeiros et al. (2016), *Spondylus limbatus* shows considerable variability in shell shape, ranging from irregularly rounded to distorted. It has an exterior surface with strong radial ribs bearing short, spathate or pointed spines (when preserved), which may decrease in size and eventually disappear; the right valve likewise exhibits concentric foliations around the attachment area, which can be rather extensive. Hinge teeth are robust and thick, brown in the left valve and white in the right valve. The adductor muscle scar varies from shallow to deep, with a ventral callus. Colourations, most evident along the broad inner margin, range from purple to orange hues. Marginal crenulations are fine and numerous. The shell can reach up to 25 cm in length.

#### Diagnosis

The hinge teeth are generally more robust, large and brown and the adductor muscle scar is deeply impressed; the spines also differ in shape and arrangement and, when the specimen retains the right valve, it may exhibit a broad attachment area (Lodeiros et al. 2016), which distinguishes it from related species. Additionally, the sculpture pattern of the ventral margin consists of fine and numerous crenulations. Dark and intense purple hues are characteristic of *Spondylus limbatus*, whereas orange and reddish tones may cause confusion with *S. crassisquama*; therefore, several morphological characters should be considered for reliable macroscopic identification.

#### Notes

*Spondylus calcifer* P. P. Carpenter, 1857 is one of the unaccepted synonyms of this species (MolluscaBase 2025).

## Analysis

### Collected samples and localities of origin

**Figure 1.**
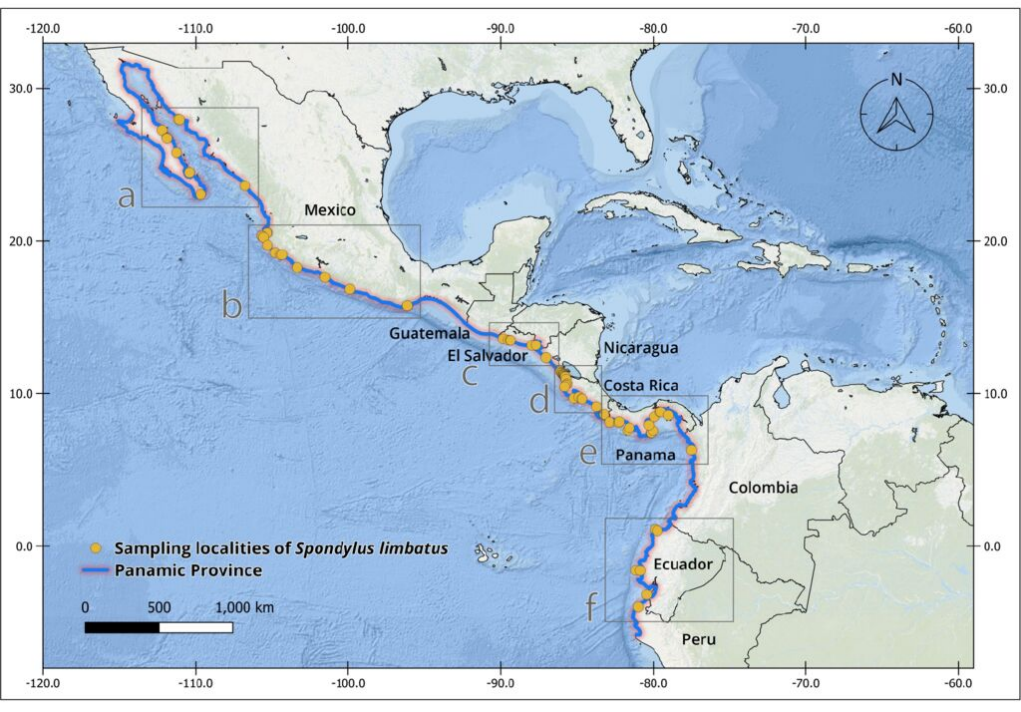
Geographical distribution of sampling localities of specimens from the Panamic Province. Maps were created in QGIS using the ESRI Ocean Basemap. Yellow dots indicate sampling localities.

The complete *Spondylus* collection from the Tropical Eastern Pacific Ocean consists of 240 specimens originating from 79 localities distributed across the Panamic Province between 28°N and 4°S. Of these, 180 individuals correspond to *Spondylus limbatus*, the most abundant species not only in terms of the number of specimens, but also in the number of localities where it was documented (n = 72/79; Tables 1, 2; Figs 1, 2). Amongst them are two individuals from Playa Corotú (Panama) and one from Bajo Seco de Súa (Ecuador), whose morphological characteristics are ambiguous, but appear to be closer to this species; therefore, they were classified as Spondylus cf. limbatus. It should also be noted that, amongst the 180 inventoried specimens of *S. limbatus*, 15 correspond to longitudinal shell sections of 15 different individuals from three Panamanian islands (Archipelago de Coiba, Isla Villa and Isla Mogo Mogo), which had previously been cut for geochemical study (Owen 2012, Carter and France 2013).

Regarding the localities, these are distributed across eight of the nine countries comprising the Panamic Province. Although fieldwork was carried out along the Guatemalan Pacific, local communities (mainly fishers and researchers) reported never having observed this species, despite the presence of small and scattered rocky outcrops along the coast. In the case of Honduras, it was not possible to establish collaborations; therefore, fieldwork could not be conducted along the Honduran coast of the Gulf of Fonseca.

**Figure 3.**
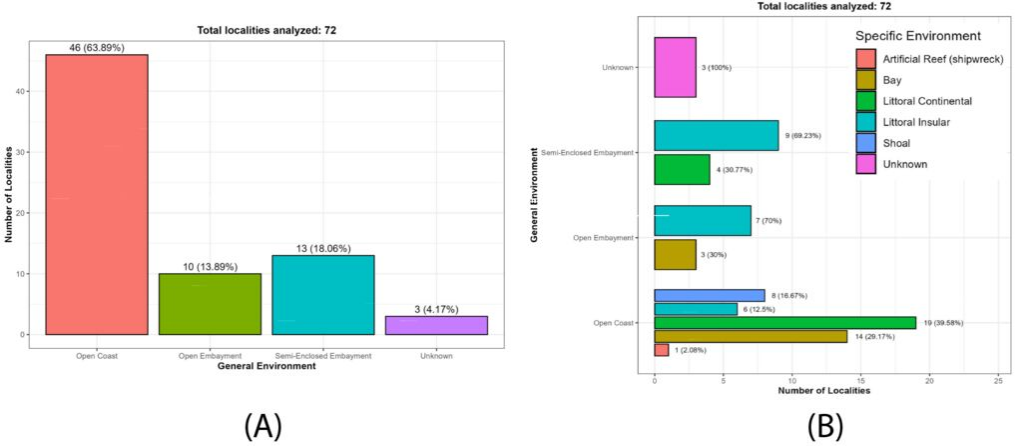
Frequency and proportional distribution of localities by general and specific environments: (**A**) Distribution of localities by general environment; (**B**) Proportional distribution of localities by general and specific environment.

In approximately 95% (n = 69/72) of the localities of origin, it was possible to characterise the general environment, which proved to be highly heterogeneous (Table 2; Fig. 3A). Environments were classified a priori as open coasts (n = 46; 63.89%), open embayment (n = 10; 13.89%) and semi-enclosed embayment (n = 13; 18.06%), which, in turn, comprise different habitat types on a smaller scale (Table 2; Fig. 3B). In addition, three localities (4.17%; Las Labradas in Mexico, Playa Corotú and El Arenal in Panama) with a total of four beach-collected samples were considered uncertain regarding the original habitat of the individuals; therefore, these were assigned to the category Unknown (Table 2; Fig. 3).

**Open coast**

Open coastal environments exhibited the greatest diversity of habitat types and localities with *Spondylus limbatus* (n = 46/72; Table 2; Fig. 3B). Open coasts are characterised by predominant exposure to the waters of the Tropical Eastern Pacific Ocean, although, in certain cases, they may also receive continental inputs. These open coasts were subdivided into littoral continental, littoral insular, bays, shallow rocky outcrops (bajos) and artificial reefs, from which the largest number of samples was obtained (n = 106/180).

Within the collection, 55 individuals originate from localities near littoral continental habitats (n = 20/72), sometimes relatively close to other environments, such as bays or capes, but without being located inside them. Examples include Campo Acosta in Mexico, Acajutla and Las Tunas in El Salvador and El Ostional de Salango in Ecuador. In addition, specimens collected at La Playita de San José del Cabo (Baja California Sur, Mexico) were reported by fishers to have been captured in the breakwater basin constructed as a jetty. Fourteen specimens were also collected from six localities situated around islands (insular shorelines), such as Isla Cocineras (also known as Roca El Roble) in Nicaragua and Isla Villa in Panama.

Furthermore, 31 specimens derive from 15 localities located within open bays, i.e. areas with wide entrances, whether large in scale — such as Bahía Banderas, which includes localities like Boca de Tomatlán or Mismaloya and Bahía de Huatulco, which includes Playa Huatulco and Playa Chahué in Mexico — or smaller in scale, such as Bahía Herradura in Costa Rica or Bahía Solano in Colombia. In addition, five specimens were collected from four shallow rocky outcrops (bajos) which may occur offshore, as in the case of Bajo El Copé (ca. 33 km) and Bajo Seco de Súa (ca. 24 km) in Ecuador or near the littoral continental, as in the outcrop associated with Bahía La Flor in Nicaragua. Finally, sunken vessels (either shipwrecks or intentionally submerged) have been colonised by various organisms, forming artificial reefs. The collection includes one locality of this type: a shipwreck off the coast of Súa, Ecuador.

**Open embayment**

These environments (mainly gulfs) are characterised by wide entrances and strong exchanges with oceanic currents, providing conditions similar to those of localities directly influenced by the open sea. However, they differ in that their interiors may include other geographic features, such as bays, capes, coves or islands, where current intensity may decrease, resulting in calmer water conditions. At least 36 individuals were collected from 10 localities situated within open gulfs (Table 2; Fig. 3B). Examples include specimens obtained along the shores of Bahía Cuajiniquil in the Gulf of Santa Elena and Bahía Culebra in the Gulf of Papagayo, both in Costa Rica. Notably, 29 individuals were collected around islands such as Granito de Oro (between this Island and Isla Ranchería) in the Archipiélago de Coiba, as well as Bolaños and San José in the Gulf of Chiriquí and Taboga and Mogo Mogo, the latter located within the Archipiélago of Las Perlas in the Gulf of Panama.

**Semi-enclosed embayment**

These environments (gulfs and bays) are characterised by relatively narrow entrances, which limit the inflow of oceanic waters compared to open embayments, resulting in less intense wave action and almost always receiving continental water inputs. Thirteen localities of this type yielded 34 individuals. As in open embayments, the samples originated from both littoral continental (3 localities, 10 shells) and insular littoral zones (9 localities, 20 shells; Table 2; Fig. 3B). Notable collection sites include Bahía Concepción in the Gulf of California, which is characterised by very calm waters and where specimens were collected around Coyote and La Liebre islands. On the continental littoral of the same Gulf, examples include Morro San Lucas and Shangri-La. A comparable semi-enclosed environment occurs at Bahía de Acapulco, where four specimens were obtained from rocky nearshore habitats at Punta Bruja (Table 2; Fig. 3B).

In addition, one specimen was obtained from Isla Meanguerita in the Gulf of Fonseca, El Salvador, while, in the Gulf of Dulce, Costa Rica, specimens were collected from Islote La Viuda and rocky outcrops off Punta Curupacha. Two localities are situated at the entrance of semi-enclosed gulfs, likely representing transitional waters between oceanic and gulf conditions: Isla Tortuga in the Gulf of Nicoya, Costa Rica and Isla Santa Clara in the Gulf of Guayaquil, Ecuador. In the latter, continental inputs are considerable, given that several rivers, including the Guayas River — the largest in Ecuador — discharges into this Gulf. However, the Island is located in the transition zone toward the open sea.

### Ecology

**Figure 4.**
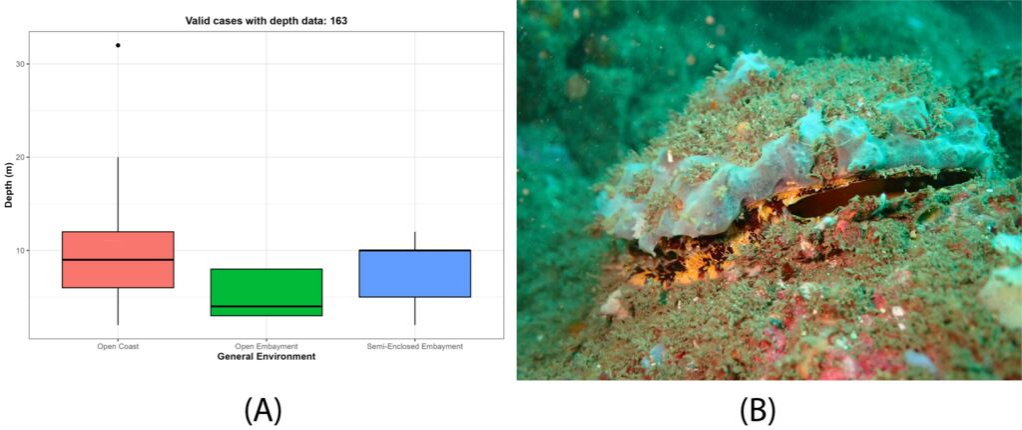
Depth patterns and habitat attachment of *Spondylus limbatus* specimens: (**A**) Specimens' collected depths by general environment; (**B**) Specimen attached to rock substrate, Acajutla, El Salvador (Photograph by A. Trejo, used with permission).

Amongst the 163 specimens with realiable depth data, most were collected in shallow nearshore waters of the intertidal zone, at depths ranging from approximately 2 m to 20 m (Fig. 4A), with a possible exception at 32 m offshore at El Palmito, Mexico. However, approximately 80% of the specimens (n = 144) were collected shallower than 12 m depth, regardless of general environment. Since these depths were estimated, based on fishermen reports, the data may vary somewhat from the actual conditions and should, therefore, be interpreted with caution. Most individuals were attached to rocky substrates (Fig. 4B), which included large boulders, slab-like rocks, cobbles and pebbles. Within these habitats, four individuals were found attached to other shells, as previously reported in literature. Three of them were using shells of *Spondylus* sp. as substrate. In one case (at Las Múcuras, Costa Rica), a double set was observed with both specimens alive, one shell cemented on to the other and the lower one was attached to rock.

Fishermen reported three specimens that were “free” amongst cobbles and pebbles on the seafloor (at Isla La Liebre, Mexico and Las Múcuras, Costa Rica) or on a shallow rocky outcrop (Bajo Seco de Súa, Ecuador). However, the right valve exhibited a small imprint, suggesting that these individuals had detached from the substrate to which they were originally attached. It should be emphasised that, although the collection localities include rocky areas, either along continental or insular littoral zones, these may, for example, alternate with other habitats such as sandy bottoms in shallow outcrops. In addition, certain localities also include coral reefs, such as Bahía Culebra in the Gulf of Papagayo or Islote La Viuda in the Gulf of Dulce, both in Costa Rica (Cortés and Jiménez 2003, Alvarado et al. 2006).

### Shell margin colour

**Figure 5.**
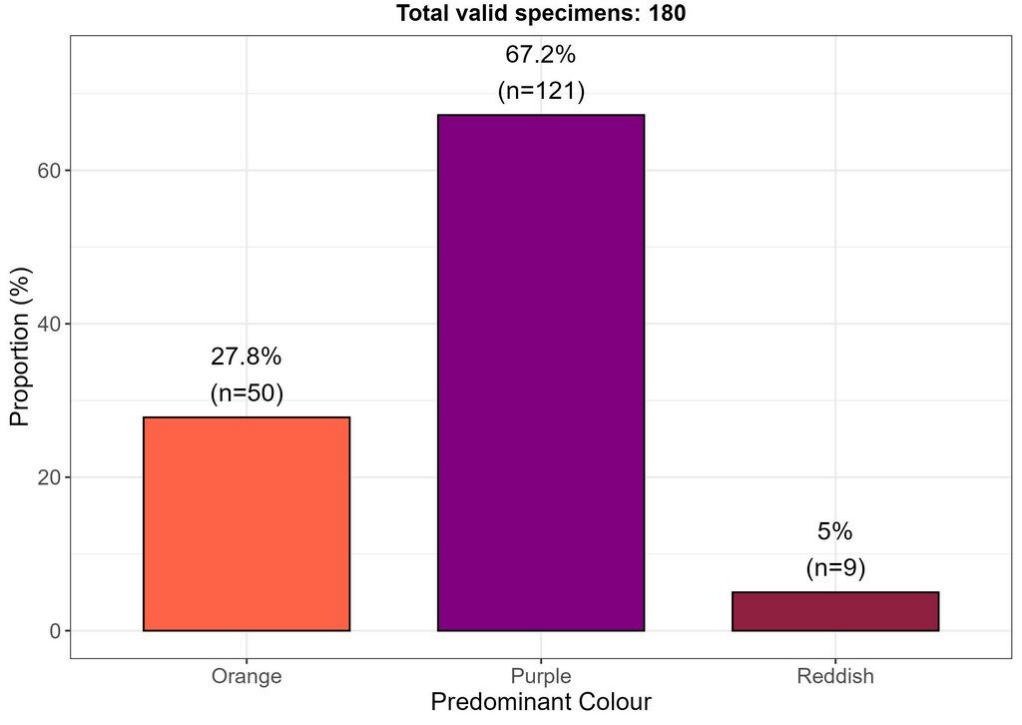
Distribution of predominant internal shell margin colours in *Spondylus limbatus* specimens from the reference collection.

**Figure 6.**
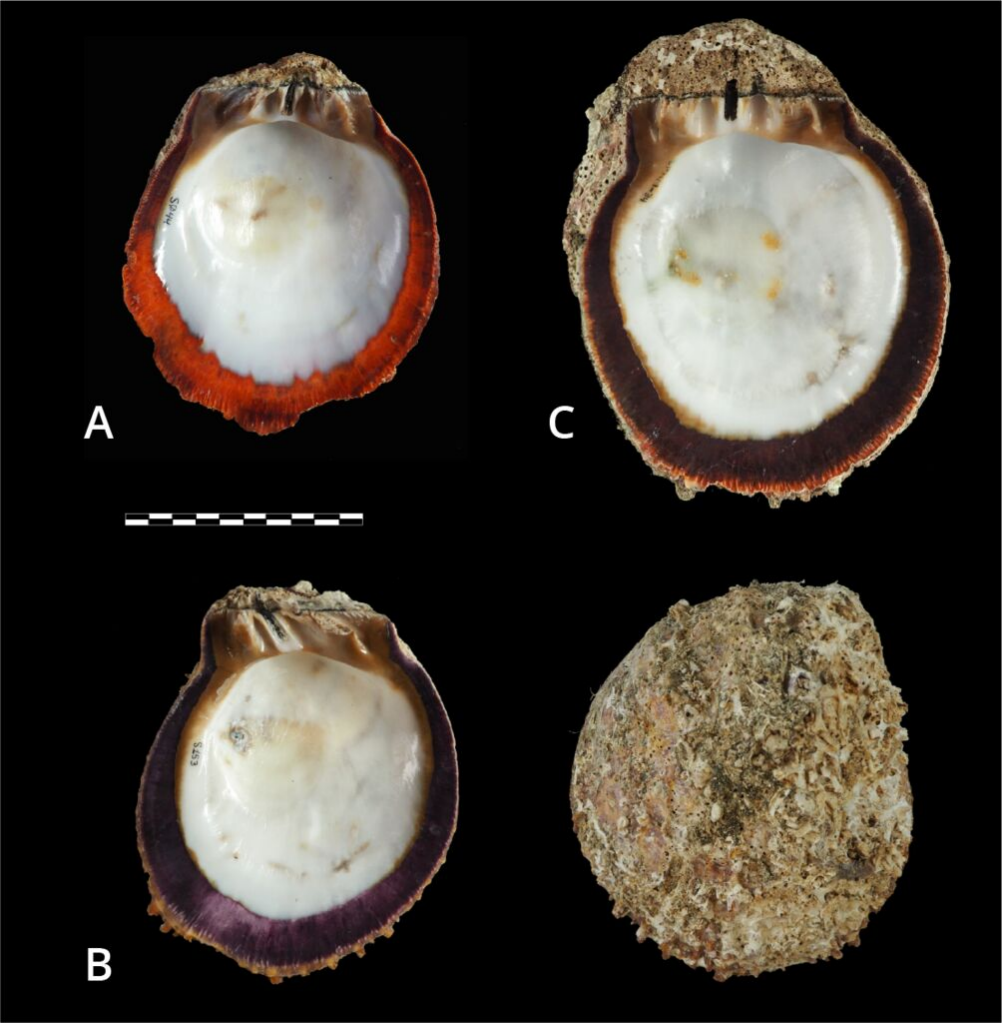
Variation in colouration along the inner margin of *Spondylus limbatus*: **(A)** orange specimen, Islote de Playa Hermosa (ID S044); (**B**) purple specimen (internal and external views), Cuajiniquil (ID S153); (**C**) reddish-brown specimen, El Ciruelo (ID S214).

Based on the colour of the internal shell margin (Figs 5, 6), purple hues were predominant across all documented environments, with a recurrence of approximately 67% (n = 121/180). These shells are the most abundant not only in the collection, but also in the natural environment, according to fishers’ reports. A smaller proportion of specimens exhibited orange hues (n = 50; 27.8%), while reddish-brown tones were scarce (n = 9; 5%). These colours may be either uniform or non-uniform along the entire internal margin (including the edge), in some cases displaying spots of varying shades of the predominant colour or even other colours, including yellow (Fig. 6B).

### Shell height

**Figure 7.**
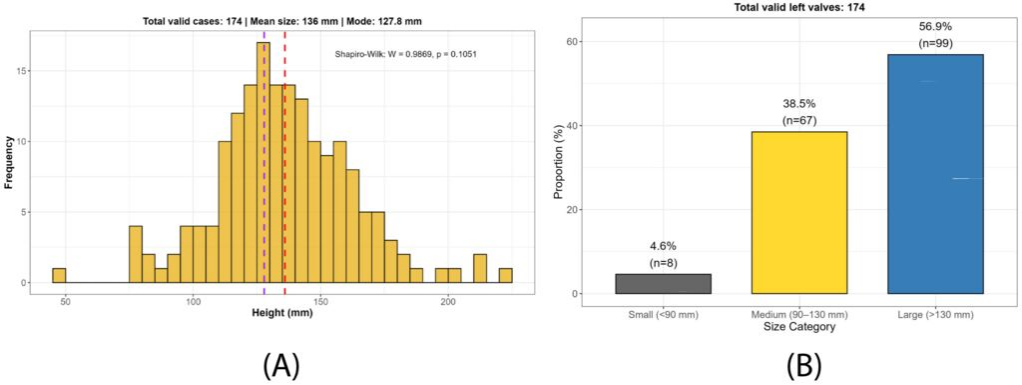
Frequency and size distribution of left valves in the collection: (**A**) Frequency distribution of left valve height in *Spondylus limbatus* specimens; (**B**) Size-class distribution of left valve specimens in the reference collection.

Regarding height, although all individuals in the collection were measured, only 174 retained the left valve (Fig. 7A). Since this valve is the most abundant, morphometric estimates were performed on it. The dataset showed a normal distribution according to the Shapiro–Wilk test (W = 0.9869; p = 0.1051). The set was divided into three size classes, with specimens larger than 130 mm being the most abundant in the collection (Fig. 7B). Height values ranged from 48.8 mm in a specimen from El Ocotal (Costa Rica) to 222.3 mm in a specimen from Bajo El Copé (Ecuador), with a mean of 136.0 mm, an estimated modal value of 127.8 mm and a standard deviation of 27.2 mm (Fig. 7A).

### Capture Methods

Specimens were collected manually by free diving, compressor-assisted diving and SCUBA diving. In the case of free diving, maximum reported depths ranged between 12 and 13 m at Boca de Tomatlán (Mexico), El Majahual and San Blas (El Salvador) and Islote de Playa Hermosa (Nicaragua). With compressor-assisted diving, greater depths were reached, including 32 m at El Palmito (Mexico).

**Figure 8.**
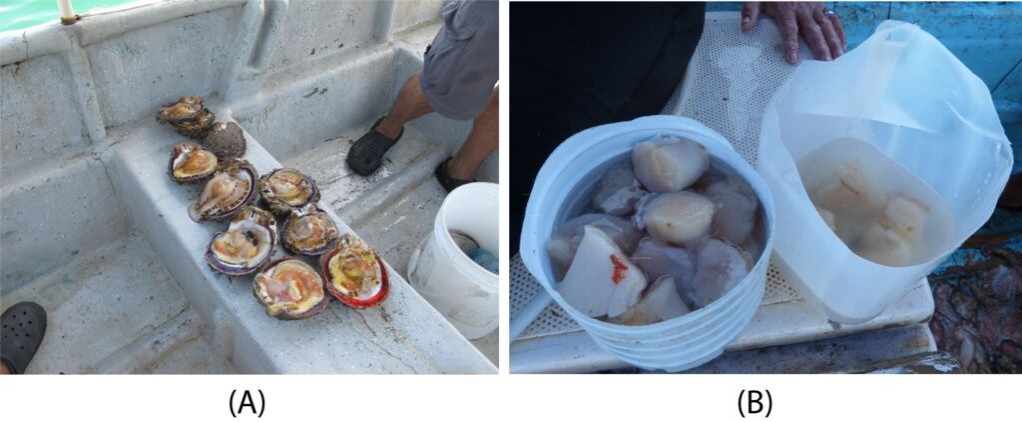
Some ways in which *Spondylus limbatus* is extracted and brought to the surface: (**A**) Left valves obtained through free diving (Isla San José, Panama); (**B**) Adductor muscle harvested only (El Palmito, Mexico).

Since *Spondylus limbatus* attached to hard substrates, divers usually extracted only the left valve (Fig. 8A), cutting the adductor muscle with a knife when the bivalve was partially open, while leaving the right valve *in situ*. This process risked fracturing the hinge teeth of the right valve, which sometimes remained attached to the left valve. Occasionally, whole individuals were removed with a chisel and hammer (~ 1 kg) from rocky shores or collected when found “free” or attached to smaller rocks or shells.

In some cases, during compressor-assisted diving, only the adductor muscle was harvested (Fig. 8B), with both valves left on the seafloor. Although uncommon, given that *Spondylus limbatus* has short spines or may lose them in adulthood, specimens collected at Súa (Ecuador) were trapped in fishing nets at depths of 11 and 14 m, at the sites known as Barco Hundido and Bajo Seco, respectively.

### Use and exploitation

**Figure 9.**
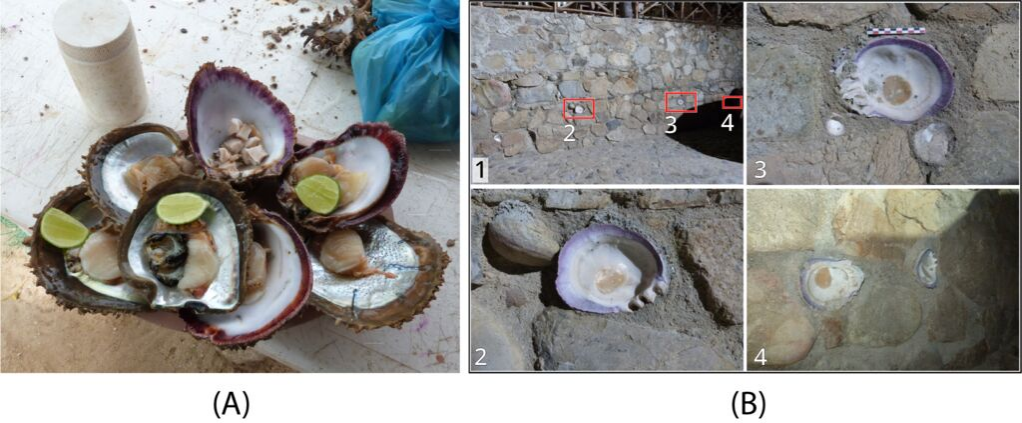
Examples of documented current uses of *Spondylus limbatus*: (**A**) In some cases, the adductor muscle of *S. limbatus* and other mollusc species, such as *Pinctada mazatlanica*, is consumed with only lemon juice (Huatulco, Mexico); (**B1**) Wall decorated with various mollusc species (Tehuamixtle, Mexico); (**B2-B4**) Details of adults *S. limbatus* specimens embedded in the wall.

The consumption of this mollusc constitutes a dietary supplement for coastal communities, but it does not represent a primary resource such as fish. Generally, only the adductor muscle is consumed (Fig. 9A) and, in rare cases, the mantle, while the rest of the organism, including the shells, is discarded in refuse dumps. Only in Mulegé (Baja California Sur, Mexico) did fishers report that, after consumption, the shells are returned to the sea on the same day of capture so that epibionts (sometimes including juvenile *Spondylus* individuals) may continue to grow. However, it was also observed that shells are sometimes used as ornaments — for example, as soap dishes, ashtrays or even embedded in architecture (Fig. 9B) — or as raw material for the manufacture of jewelry.

## Discussion

### New data records

The reference collection comprised 240 specimens of Spondylidae, of which 180 were identified as *Spondylus limbatus*. This modern dataset provides valuable information on the distribution, ecology and other aspects of a species considered of interest to fisheries in the region (Barraza 2006, Holguín Quiñones 2006, Farías-Tafolla et al. 2022, INCOPESCA 2023). Specimens originated from 72 localities in eight countries, thereby complementing the existing distributional records (Skoglund and Mulliner 1996, Coan and Valentich-Scott 2012, Lodeiros et al. 2016, Carter 2022).

The collections confirmed the persistence of populations previously reported in literature, such as Bahía Concepción (e.g. Isla Coyote) in Mexico (Skoglund and Mulliner 1996), Bahía Solano in Colombia (Bernard et al. 1991) and El Ostional in Salango, Ecuador (Aguilar et al. 2013). In addition, approximately 40 new localities were documented (Table 1; Figs 1, 2), filling important gaps in regions where *S. limbatus* had been poorly recorded, such as Jalisco and Colima in Mexico (Holguín Quiñones 2006, Landa-Jaime et al. 2013, Ríos Jara et al. 2017), El Salvador (Barraza 2006, Barraza 2018, Barraza 2021), Nicaragua (López de la Fuente and Urcuyo Ramos 2008) and Panama (Skoglund and Mulliner 1996, Carter and France 2013, Carter 2022), amongst others. These findings significantly expand the known distribution of the species, which had previously been fragmentary and suggested a locally scarce presence. A notable case is the locality popularly known as Barco Hundido in Súa (0°59'55"N, 79°44'41"W), a shipwreck situated approximately 14 km west of a site previously reported as “El Barco” (Aguilar et al. 2013). This finding highlights the need for more detailed exploration and documentation of shipwrecks that have become artificial reefs.

Taken together, this reference collection demonstrates that, beyond the well-known concentration and exploitation areas in Baja California Sur, Acapulco and Ecuador (Marcos and Norton 1981, Cudney-Bueno and Rowell 2008, Mackensen 2013, Carter 2022), other regions, such as the coasts of Jalisco and Colima in Mexico, western El Salvador and the south-western Nicaragua–Panama corridor, may also represent additional concentration zones for the species. This is supported by the fact that these areas’ geographic and environmental conditions are favourable for the development of benthic species such as *Spondylus* (Alvarado et al. 2006, Ríos Jara et al. 2016, Ríos Jara et al. 2017). In addition, although population density estimates are still lacking, the records suggest that the distribution and abundance of *S. limbatus* in the region has been under-documented and may have been underestimated.

### Habitat and ecology

The results confirm that *Spondylus limbatus* occurs on rocky and/or coral formations — such as those documented in Bahía Culebra in the Gulf of Papagayo and Islote La Viuda in the Gulf of Dulce, Costa Rica (Cortés and Jiménez 2003, Alvarado et al. 2006) — in shallow waters, as previously reported for this and other species of the family Spondylidae (Keen 1971, Skoglund and Mulliner 1996, Cudney-Bueno and Rowell 2008, Coan and Valentich-Scott 2012, Lodeiros et al. 2016). This habitat is essential, as it provides hard substrates, such as rocks, cobbles and shells, which are required for the settlement and development of the species. Such environments are typically found along open coasts and in embayments — both open and semi-enclosed — such as gulfs and bays.

A particularly relevant finding was the presence of *S. limbatus* individuals attached to the shells of dead conspecifics. This observation validates local fishers’ reports of returning shells to the sea after consumption, thereby promoting the continued growth of organisms attached to the outer surface of the valves, potentially including juvenile *Spondylus*. This interpretation is consistent with the aquaculture study of Lodeiros et al. (2016), who reported that, after reproduction, larvae settle on hard substrates, such as other shells, which may explain the benefit of this practice. Although no studies have quantified the frequency of juveniles attached to conspecific shells, this practice of returning shells could, to some extent, help mitigate the decline of populations that continue to be exploited for their adductor muscle (Holguín Quiñones 2006, Lodeiros et al. 2016).

Although fishers in some localities reported a few apparently “free-living” individuals, the presence of small attachment scars on the right valve suggests that these specimens had become detached, either naturally or through anthropogenic disturbance, from the substrates to which they had originally been fixed.

### Exploitation and Sustainability Considerations

*Spondylus limbatus* is classified as a fisheries resource in many countries, including El Salvador (Barraza 2006), Nicaragua (Instituto Nicaragüense de la Pesca y Acuicultura 2019) and Costa Rica (INCOPESCA 2023), where its local commercial exploitation is permitted under different legislative frameworks. In Mexico and Ecuador, the species is subject to permanent fishing bans, with some exceptions (Subsecretaría de Recursos Pesqueros, Ecuador 2009, Secretaría de Medio Ambiente y Recursos Naturales 2010).

The present study did not aim to quantitatively assess the impact or sustainability of exploitation, which would require specific studies of population density and fishing effort. Available studies in the region are scarce and largely restricted to Baja California Sur (Mexico) and a few localities along the Ecuadorian coast (Mackensen et al. 2011, Aguilar et al. 2013, Villalejo-Fuerte et al. 2020), where reported population density indices are low. This limitation hinders the evaluation of exploitation levels across much of the species’ range. Such data gaps may partly reflect a broader tendency for conservation and research efforts to focus on oyster species of higher economic value, such as *Pinctada mazatlanica* in Panama (Galtsoff 1950, Cipriani et al. 2008, Spalding and Mellado 2018), leaving *Spondylus* comparatively understudied. Nevertheless, testimonies from fishers in Jalisco (Mexico), El Salvador and Ecuador suggest that the species was more abundant in shallow waters (< 5–10 m) prior to the 1990s, which may indicate a local decline.

In this context, potential conservation measures could include restricting exploitation to periods outside the reproductive season, limiting harvests to adult individuals (> 90 mm shell height; Villalejo-Fuerte et al. 2020, Villalejo-Fuerte et al. 2023), given that juvenile extraction has been observed in some localities (e.g. Acapulco and Bahía de Huatulco, Mexico) and returning shells to the sea immediately after consumption. However, the effectiveness of such measures would depend on the implementation of targeted studies and management actions across the species range.

### Potential environmental constraints

The apparent absence of *Spondylus limbatus* along the Guatemalan coast and adjacent areas, as identified by Carter (2022) from a bibliographic review, is likely related to biogeographic factors. Local inhabitants in this region did not recognise the presence of *S. limbatus*, reinforcing this observation. Unlike other areas characterised by gulfs, bays with cliffs or rocky formations, the Guatemalan coast is predominantly a broad, rectilinear plain with volcanic black-sand beaches (Fonseca E. and Arrivillaga 2003, Boix Morán et al. 2011, SEGEPLAN 2011, Dávila Pérez et al. 2014). This coastal plain is interrupted by river mouths, sandbars, estuaries, channels and coastal lagoons (Melendreras Wilkin 2008, Estrada et al. 2024). The seafloor consists mainly of sand and clayey silt, with little relief or structural complexity (Melendreras Wilkin 2008). These characteristics make the occurrence and exploitation of species, such as *S. limbatus*, unlikely in this region. Although some isolated corals occur on hard substrates (Fonseca E. and Arrivillaga 2003), as well as artificial structures, such as the docks at Puerto Quetzal (Portuaria Quetzal 2017) or artificial reefs deployed in recent years (CONAP & MARN 2009, Morales Díaz 2015), no reliable records of *S. limbatus* have been confirmed to date, despite a mention by Pérez Ramírez (2006).

A similar apparent absence was noted in gulf or bay environments strongly influenced by freshwater input, such as Bahía San Miguel in Panama, where the Tuira River discharges and the Gulf of Guayaquil in Ecuador, influenced by the Guayas River. These results reinforce the ecological association of *S. limbatus* with hard-substrate habitats removed from the direct influence of substantial fluvial discharge.

### Depth

Specimens were collected at depths ranging from 2 m to 32 m, although most were obtained at less than 12 m, corroborating previous observations by Aguilar et al. (2013) and Carter (2022). In the present study, the relative abundance at these shallow depths may be partly explained by the fact that many specimens were collected through free diving, a technique limited in both depth and bottom time. Collecting individuals at greater depths generally requires the use of alternative diving methods. Population density studies in the Gulf of California have similarly reported considerable abundance of *S. limbatus* at approximately 10 m depth (Villalejo-Fuerte et al. 2020). On the other hand, *Spondylus* living at shallow depths (< 2 m) may have been preferentially harvested and have, therefore, become rare in infralittoral waters.

### Height and colour variation

The analysis of shell height in 174 specimens with preserved left valves showed a range between 48.8 mm and 222.3 mm (mean = 136 mm; mode = 127.8 mm), which overall exceeds the values reported for the Gulf of California by Villalejo-Fuerte et al. (2023), who documented sizes between 8.2 and 167 mm (mean = 126 mm). Nearly all individuals measured more than 90 mm in height (Fig. 7), indicating that they correspond to adult specimens (Cudney-Bueno and Rowell 2008, Mackensen and Brey 2013). However, because of the challenges in establishing parameters related to asymptotic shell height for this species (Villalejo-Fuerte et al. 2023), it remains difficult to reliably correlate shell size with individual age. Nevertheless, these results provide a regional overview of the sizes currently exploited, which are also closely linked to resource availability.

With respect to colouration, the predominance of purple hues is characteristic of this species and consistent with previous reports (Keen 1971, Skoglund and Mulliner 1996, Coan and Valentich-Scott 2012, Villalejo-Fuerte et al. 2015). However, orange-coloured individuals were also recorded in several localities, occurring in the same environments and under the same conditions as purple- and reddish-toned shells (Fig. 6). In some cases, specimens exhibited one orange valve and one purple valve or even a single valve displaying both hues simultaneously. The factors underlying this chromatic variation remain unknown and represent a promising avenue for future research.

### Implications

One of the potential biases of this study lies in the sampling strategy, as an average of only four specimens was recovered per locality. Due to the small number of shells collected and the selection method, this sample does not provide an exhaustive representation of the diversity that may exist at each site, particularly in those where only a single specimen was obtained (e.g. Isla Meanguerita in El Salvador or Ocotal in Costa Rica). Nevertheless, the broad variety of localities and environments represented by the 180 *Spondylus limbatus* specimens included in the reference collection offers a useful regional overview of the species. It should also be considered that, in each locality, larger individuals or specimens with morphological and chromatic variations different from those documented here may occur.

In addition, ethnohistorical sources have suggested possible pre-Hispanic harvesting zones along the Guerrero coast, Mexico (Temple Sánchez-Garavito and Velázquez Castro 2003). However, only samples from El Yunque, a shoal south of Zihuatanejo and from Acapulco (Punta Bruja) — the latter previously documented (Gutiérrez Zavala and Cabera Mancilla 2012, Castro-Mondragón et al. 2016) — were recovered. This limits the extent to which the present collection can be used to verify such hypotheses in future studies.

Despite these limitations, the collection has relevance not only for archaeomalacological research, but also for improving our understanding of the distribution and ecology of the species, as previously discussed. Moreover, it provides a means to document the environmental conditions in which these individuals lived and opens the possibility of establishing future geochemical datasets that may be applied in other lines of research. In this sense, the collection offers opportunities for interdisciplinary studies on marine bivalves of the family Spondylidae, including those related to conservation and the sustainable management of this fishery resource — provided that ongoing over-exploitation does not cause irreversible damage to its populations.

## Supplementary Material

XML Treatment for Spondylus limbatus

## Figures and Tables

**Figure 2a.**
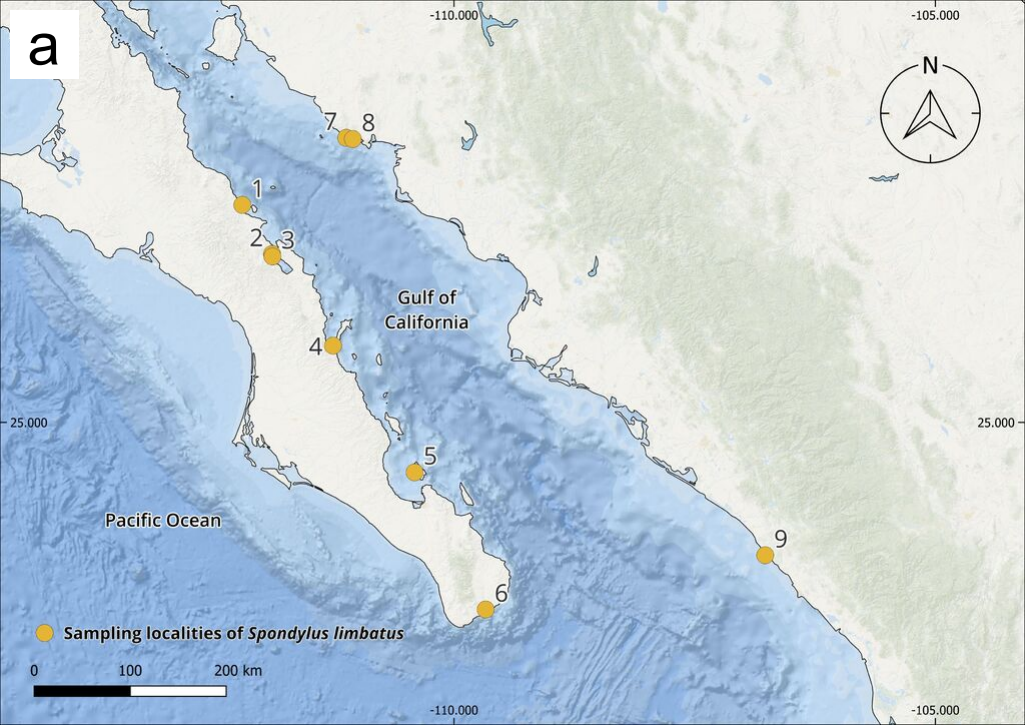
Gulf of California;

**Figure 2b.**
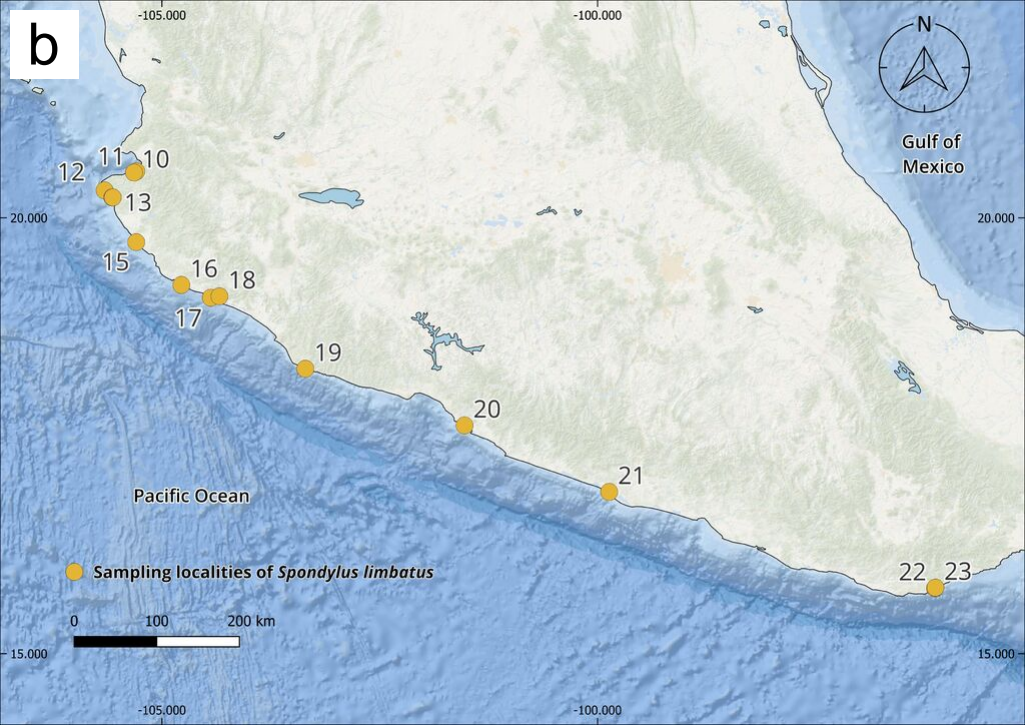
Central Mexico;

**Figure 2c.**
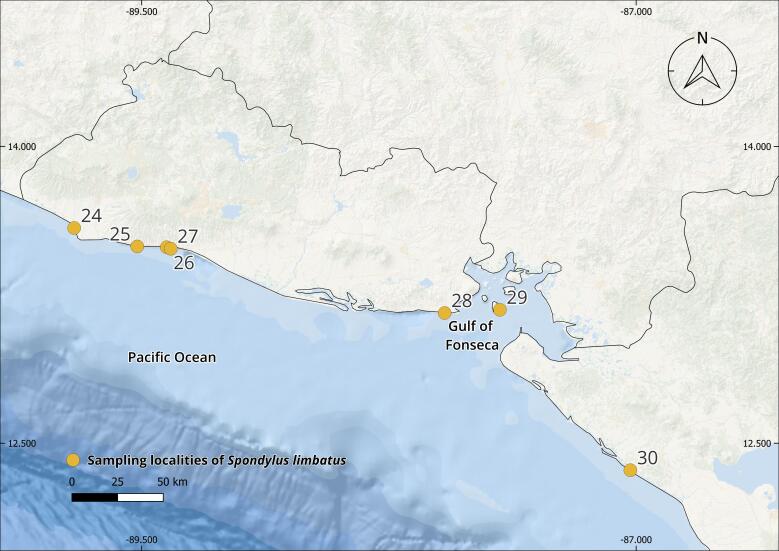
El Salvador-northwestern Nicaragua;

**Figure 2d.**
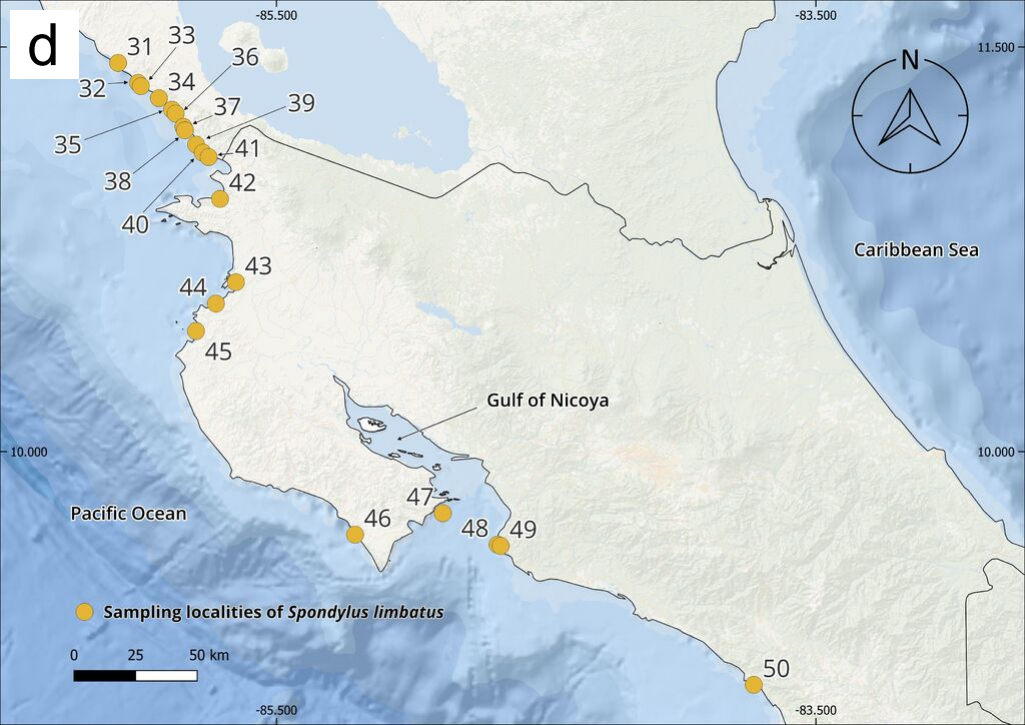
South-western Nicaragua-Costa Rica;

**Figure 2e.**
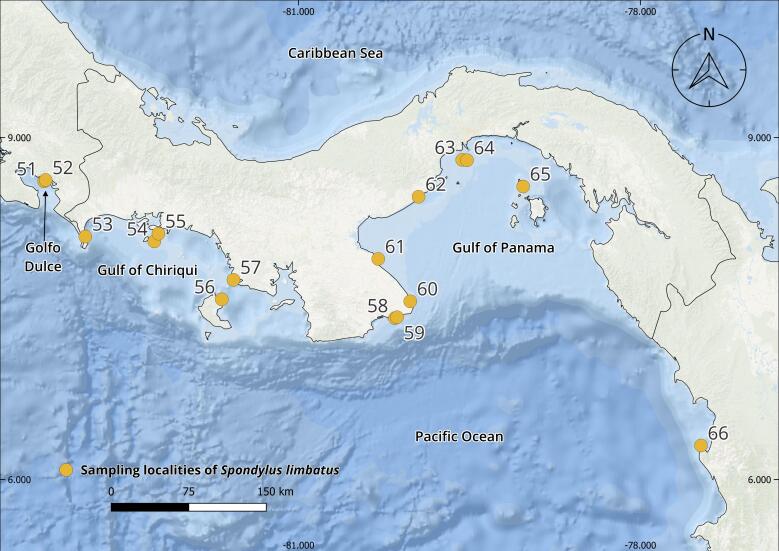
Southern Costa Rica-northwestern Colombia;

**Figure 2f.**
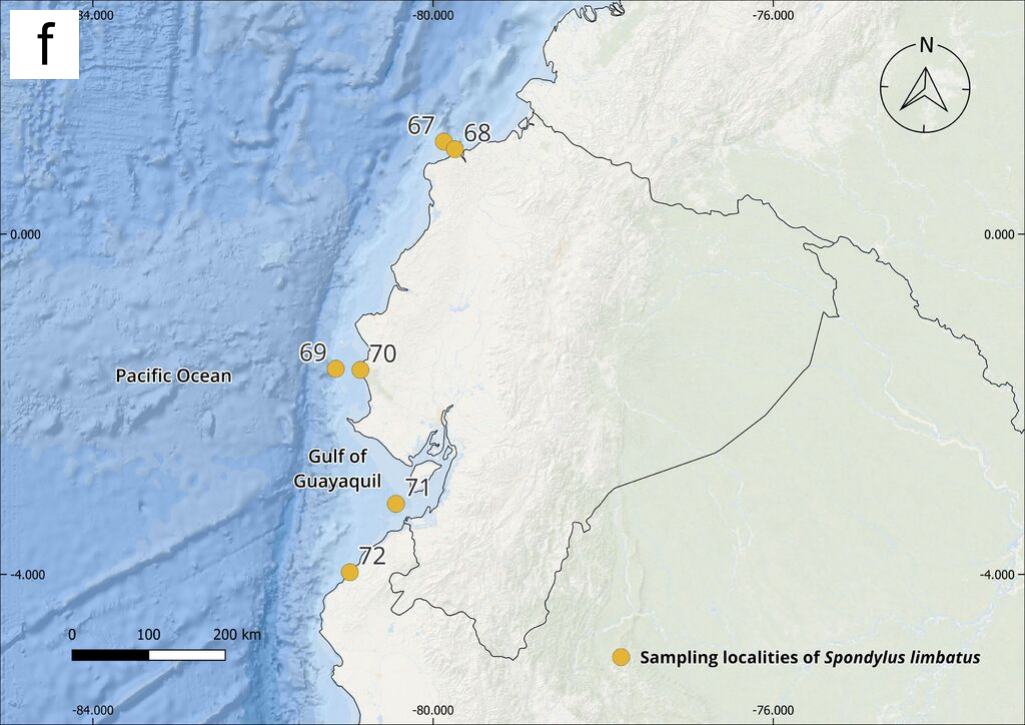
Ecuador and northern Peru.

**Table 1.** Localities of origin of *Spondylus limbatus* samples and number of specimens collected (Figs 1, 2). * New locality record.

**Country**	**State/Dept./Province: Localities^[number of specimens collected per locality]^**	**n = total country**	%
Mexico (Fig. 2a, b)	*Baja California Sur*: 1. *Morro San Lucas^1^, 2. Isla La Liebre^2^, 3. Isla El Coyote^3^, 4. Isla Danzante^1^, 5. *Isla Ballena^1^, 6. *La Playa de San José del Cabo^3^ *Sonora*: 7. *Isla El Venado^1^, 8. Shangri-la^3^ *Sinaloa*: 9. Las Labradas^1^ *Jalisco*: 10. *Mismaloya^1^, 11. *Boca de Tomatlán^1^, 12. *Playa Cucharas^1^, 13. *Tehuamixtle^4^, 14. *Hakuna Matata^1^, 15. *Campo Acosta^3^, 16. *El Palmito^4^ *Colima*: 17. *Palo Alto^3^, 18. Punta de las Hadas^2^ *Michoacán*: 19. *Maruata Viejo^3^ *Guerrero*: 20. *El Yunque^4^, 21. Punta Bruja^4^ *Oaxaca*: 22. Playa Huatulco^4^, 23. Playa Chahué^3^	56	31.1
El Salvador (Fig. 2c)	*Sonsonate*: 24. *Acajutla^3^ *La Libertad*: 25. *La Perla^1^, 26. *El Majahual^4^, 27. *San Blas^5^ *La Unión*: 28. *Las Tunas^1^, 29. *Isla Meanguerita^1^	15	8.3
Nicaragua (Fig. 2c, d)	*León*: 30. *Las Peñitas^1^ *Rivas*: 31. *Playa Santana^1^, 32. *Playa Manzanillo^1^, 33. *Isla La Vieja^2^, 34. *Playa Blanca^1^, 35. *Bahía de Nacascolo^1^, 36. San Juan del Sur^1^, 37. *Islote de Playa Hermosa^2^, 38. *Isla Las Palomas^1^, 39. Bajo de Bahía La Flor^1^, 40. *Isla Cocineras^3^, 41. *El Ostional^2^	17	9.4
Costa Rica (Fig. 2d, e)	*Guanacaste*: 42. Cuajiniquil^4^, 43. Bahía Culebra^2^, 44. Ocotal^1^, 45. *Las Mucuras^3^ *Puntarenas*: 46. *Manzanillo^3^, 47. Isla Tortuga^3^, 48. *Punta Leona^4^, 49. Bahía Herradura^1^, 50. *Isla Ballena^1^, 51. Islote La Viuda^4^, 52. Punta Curupacha^4^	30	16.7
Panama (Fig. 2e)	*Chiriquí*: 53. *Playa Corotú^2^, 54. *Isla Bolaños^2^, 55. *Isla San José^6^ *Veraguas*: 56. Isla Granito de Oro (Archipiélago de Coiba)^5^, 57. *Isla Canales de Tierra^4^ *Los Santos*: 58. *El Calabacito^4^, 59. *El Ciruelo^2^, 60. Playa El Arenal^1^, 61. Isla Villa^5^ *Panamá Oeste*: 62. *San Carlos^1^ *Panamá*: 63. Isla Taboga^3^, 64. *Isla Taboguilla^4^, 65. Isla Mogo Mogo^5^	44	24.5
Colombia (Fig. 2e)	*Chocó*: 66. Bahía Solano^3^	3	1.7
Ecuador (Fig. 2f)	*Esmeraldas*: Súa (67. Bajo Seco^1^; 68. *Barco Hundido^1^) *Manabí*: 69. Bajo El Copé^1^, 70. El Ostional de Salango^6^ *El Oro*: 71. Isla Santa Clara^4^	13	7.2
Peru (Fig. 2f)	*Tumbes*: 72. Punta Sal^2^	2	1.1
**Total number of specimens in the Reference Collection**		**180**	**100**%

**Table 2.** Distribution of sampled localities by general and specific environment.

General environment	Specific environment	Country and Locality
Open Coast	Bay	*Mexico*: 10. Mismaloya, 11. Boca de Tomatlán, 16. El Palmito, 18. Punta de las Hadas, 19. Maruata Viejo, 22. Playa Huatulco, 23. Playa Chahué *Nicaragua*: 32. Playa Manzanillo, 34. Playa Blanca, 35. Bahía de Nacascolo, 36. San Juan del Sur, 41. El Ostional *Costa Rica*: 45. Las Múcuras, 49. Bahía Herradura *Colombia*: 66. Bahía Solano
	Littoral Continental	*Mexico*: 6. La Playa de San José del Cabo, 12. Playa Cucharas, 13. Tehuamixtle, 14. Hakuna Matata, 15. Campo Acosta, 17. Palo Alto, 20. El Yunque *El Salvador*: 24. Acajutla, 25. La Perla, 26. El Majahual, 27. San Blas, 28. Las Tunas *Nicaragua*: 30. Las Peñitas, 31. Playa Santana *Costa Rica*: 46. Manzanillo, 48. Punta Leona *Panama*: 58. El Calabacito, 59. El Ciruelo, 62. San Carlos *Ecuador*: 70. El Ostional de Salango
	Littoral Insular	*Nicaragua*: 33. Isla La Vieja, 37. Islote de Playa Hermosa, 38. Isla de las Palomas, 40. Isla Cocineras *Costa Rica*: 50. Isla Ballena *Panama*: 61. Isla Villa
	Shoal	*Nicaragua*: 39. Bajo de Bahía La Flor *Ecuador*: 67. Bajo Seco de Súa, 69. Bajo El Copé *Peru*: 72. Punta Sal
	Shipwreck	*Ecuador*: 68. Barco Hundido de Súa
Open Embayment	Bay	*Costa Rica*: 42. Cuajiniquil, 43. Bahía Culebra, 44. Ocotal
	Littoral Insular	*Panama*: 54. Isla Bolaños, 55. Isla San José, 56. Isla Granito de Oro (Archipiélago de Coiba), 57. Isla Canales de Tierra, 63. Isla Taboga, 64. Isla Taboguilla, 65. Isla Mogo Mogo (Archipiélago de las Perlas)
Semi-Enclosed Embayment	Littoral Continental	*Mexico*: 1. Morro San Lucas, 8. Sangri-La, 21. Punta Bruja *Costa Rica*: 52. Punta Curupacha
	Littoral Insular	*Mexico*: 2. Isla La Liebre, 3. Isla Coyote, 4. Isla Danzante, 5. Isla Ballena, 7. Isla El Venado *El Salvador*: 29. Isla Meanguerita *Costa Rica*: 47. Isla Tortuga, 51. Islote La Viuda *Ecuador*: 71. Isla Santa Clara
Unknown		*Mexico*: 9. Las Labradas *Panama*: 53. Playa Corotú, 60. Playa El Arenal
